# Structural and thermodynamic insight into phenylalanine hydroxylase from the human pathogen *Legionella pneumophila*^☆^

**DOI:** 10.1016/j.fob.2013.08.006

**Published:** 2013-08-19

**Authors:** Hanna-Kirsti S. Leiros, Marte Innselset Flydal, Aurora Martinez

**Affiliations:** a The Norwegian Structural Biology Centre (NorStruct), Department of Chemistry, University of Tromsø, N-9037 Tromsø, Norway; b Department of Biomedicine, University of Bergen, Jonas Lies vei 91, 5009 Bergen, Norway

**Keywords:** Pyomelanin synthesis, Thermostability, Substrate specificity, Aggregation, Salt induced aggregation, Pathogen

## Abstract

Phenylalanine hydroxylase from *Legionella pneumophila* (lpPAH) has a major functional role in the synthesis of the pigment pyomelanin, which is a potential virulence factor. We present here the crystal structure of lpPAH, which is a dimeric enzyme that shows high thermostability, with a midpoint denaturation temperature of 79 °C, and low substrate affinity. The structure revealed a dimerization motif that includes ionic interactions and a hydrophobic core, composed of both β-structure and a C-terminal region, with the specific residues (P255, P256, Y257 and F258) interacting with the same residues from the adjacent subunit within the dimer. This unique dimerization interface, together with a number of aromatic clusters, appears to contribute to the high thermal stability of lpPAH. The crystal structure also explains the increased aggregation of the enzyme in the presence of salt. Moreover, the low affinity for substrate l-Phe could be explained from three consecutive glycine residues (G181, 182, 183) located at the substrate-binding site. This is the first structure of a dimeric bacterial PAH and provides a framework for interpreting the molecular and kinetic properties of lpPAH and for further investigating the regulation of the enzyme.

## Highlights

• The structure *Legionella pneumophila* PAH (lpPAH) has been resolved
• The *T_m_ of lpPAH at* 79 °C is explained by structure
• The unique dimer interface of lpPAH comprises aromatic and ionic interactions
• Tyr257 seems important for dimerization
• This is the first structure of a dimeric bacterial PAH

## Graphical abstract

**Figure:**
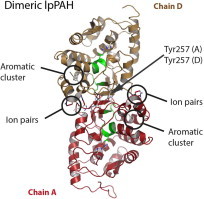

## 1 Introduction

The gram-negative bacterium *Legionella pneumophila* belongs to the γ-proteobacteria. In nature, *L. pneumophila* is an inhabitant of warm freshwater habitats where its multiplication is mainly restricted to intracellular niches inside amoebal hosts and after infection it continues this intracellular life-style within the human host by multiplying inside macrophages [1]. Humans are a dead-end host for this pathogen, but it still causes outbreaks of Legionnaires’ disease, a potentially fatal form of pneumonia, when it multiplies in warm, stagnant water that are spread in aerosols through human-made installations such as fountains and showers [2–4]. In such aqueous environments *L. pneumophila* grows well at temperatures in the range 20–48 °C, but its tolerance to higher temperatures can lead to regrowth after heat-disinfection [5]. The number of reported cases of Legionnaires’ disease has increased in the last decade [6], making eradication of the bacterium from infection sources an important task. Thus, there is a need for research on possible targets, notably on putative virulence factors.

*L. pneumophila* is one of several known pathogenic bacteria whose genomes encode a phenylalanine hydroxylase (PAH). PAH is the enzyme that catalyzes the hydroxylation of l-phenylalanine (l-Phe) to l-tyrosine (l-Tyr) using non-heme Fe(II) and tetrahydrobiopterin (BH_4_) as cofactors, and dioxygen as additional substrate [7]. In mammals, PAH has a major catabolic role and its dysfunction is associated to deleterious hyperphenylalaninemia and phenylketonuria, whereas as we have recently shown [8] *L. pneumophila* PAH (lpPAH) has an important role in the growth of the bacterium in media deficient in l-Tyr and in the synthesis of a brownish pigment called pyomelanin. Like some of the other bacteria that encode a PAH, *L. pneumophila* produces and excretes pyomelanin when l-Phe or l-Tyr is available [9]. Pyomelanin is produced via the catabolism of l-Phe/l-Tyr when an intermediate of the pathway, homogentisate, accumulates and subsequently autooxidises and polymerises. In *L. pneumophila*, the pyomelanin has ferric reductase properties, scavenging and reducing extracellular iron [10]. Iron is an essential metal for both human host and bacterial pathogens, among other as cofactor for many enzymes, including PAH. Iron is indeed critical for intracellular infection by *L. pneumophila* [11] and production of pyomelanin is one of several strategies for iron assimilation [10]. Molecular and enzyme kinetic characterization has shown that lpPAH is well suited for catalysis at human body temperature, revealing a maximum activity around 40 °C and very high specific activity compared to other eukaryote and prokaryote PAHs [8,12–16]. The enzyme also presented high *K*_m_ values for substrate and cofactor BH_4_ (735 ± 50 μM and 125 ± 25 μM, respectively) [8], and the low affinity for l-Phe has been interpreted as a plausible regulatory mechanism to preserve threshold amounts of the substrate [7]. Similarly to all other PAHs, the activity of lpPAH is totally dependent upon a non-heme ferrous ion, but – in contrast to all other studied PAHs which are iron-bound tetramers (eukaryotes) or monomers (bacteria) [7,15,17] – lpPAH is isolated as an iron-free (apo) homodimer when expressed recombinantly [8]. The estimated hydrodynamic diameter of apo-lpPAH dimer (8.1 ± 0.1 nm) and its surprisingly high thermal stability (midpoint denaturation temperature (*T*_m_) = 79 ± 0.5 °C) is only slightly increased by Fe incorporation [8].

The high thermal stability of lpPAH might be important for preservation of the enzyme at temperatures where *L. pneumophila* survives in a dormant, non-replicative state [18] and, based on a crucial role of the enzyme in the synthesis of pyomelanin, we set off to characterize the structure–function–stability relationships in dimeric lpPAH. The recombinant enzyme was crystallized and its structure has been analyzed and compared with that of PAHs from mesophilic *Chromobacterium violaceum* (cvPAH; optimum temperature for growth, 25 °C) and psychrophilic *Colwellia psychrerythraea 34H* (cpPAH; optimum temperature for growth, 10 °C) bacteria, as well as with the human enzyme (hPAH; optimum temperature, 37 °C). The structural features identify a unique dimerization motif and aid to explain the particular structural determinants for thermal adaptation of lpPAH, as well as its low affinity for l-Phe and BH_4_ [8].

## 2 Results and discussion

### 2.1 Refinement and overall structure

The crystallization of lpPAH was challenging since the crystals grew as plates with some disorder found in the final models, and due to the low symmetry spacegroup *P*2_1_ a decent rotation range of X-ray data was needed to obtain more that 90% completeness. The best crystal diffracted to 2.5 Å (Table 1) and in the observed electron density maps the dimeric lpPAH structure is clearly defined, in particular for chains A and B. Thus, the current model gives good insight into the lpPAH structure as described below.

**Fig. 1:**
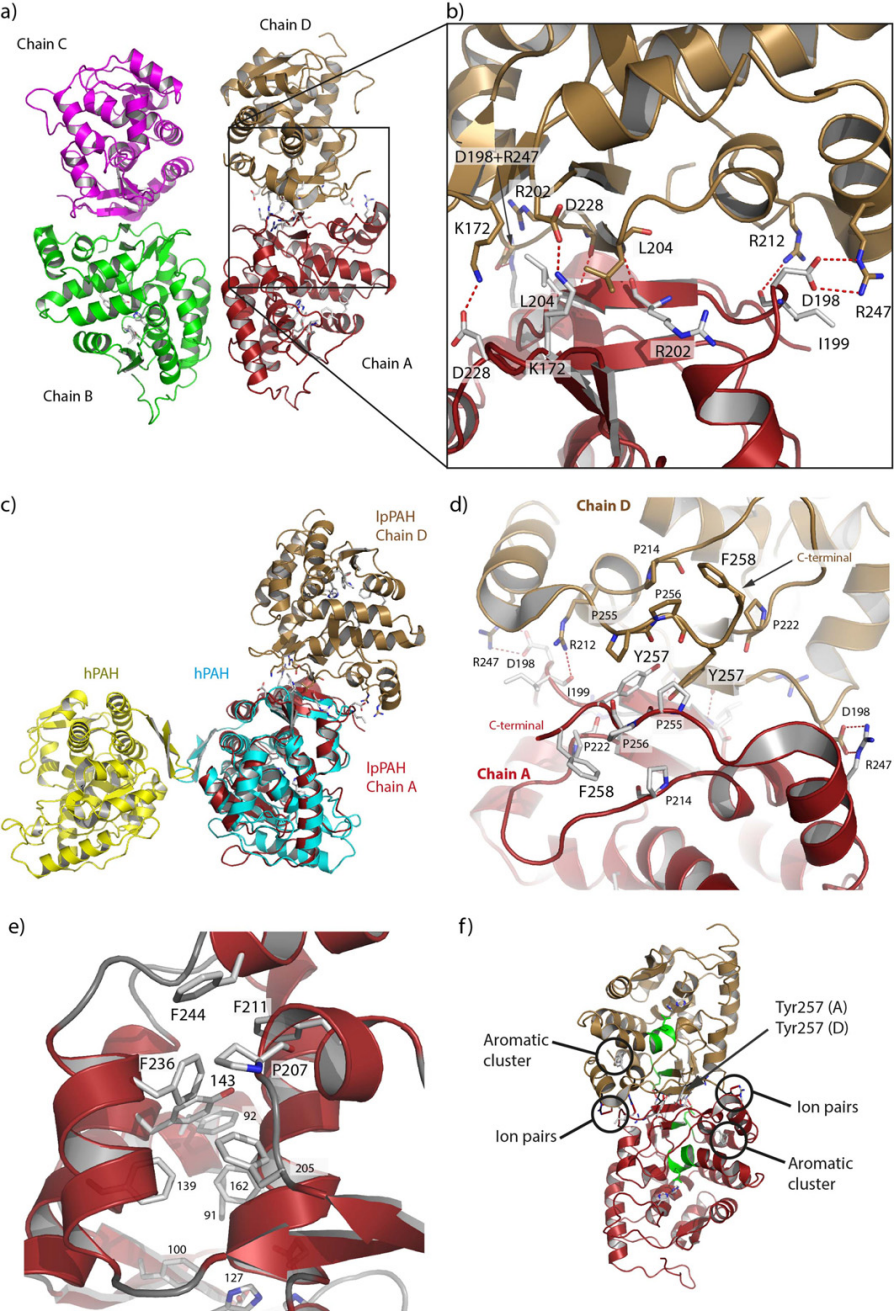
Overall structure of lpPAH. (a) The two lpPAH dimers (chains A–D) in the asymmetric unit. (b) Ionic interactions and (d) stacking interactions at the dimer interface of chains A (red) and D (sand). (c) Superimposition of hPAH truncated dimer (subunits in yellow and cyan; PDB 1PAH [21]) onto the lpPAH dimer (chains A and D in red and sand, respectively). (e) A large aromatic cluster, with the unique lpPAH residues Phe211, Phe236 and Phe244. (f) Residues 163–168 and 225–229 (motifs in green) predicted to be prone to β-aggregation by the TANGO-algorithm, surrounded by ion pairs and aromatic clusters [38] in one lpPAH dimer. (For interpretation of the references to colour in this figure legend, the reader is referred to the web version of this article.)

**Fig. 2:**
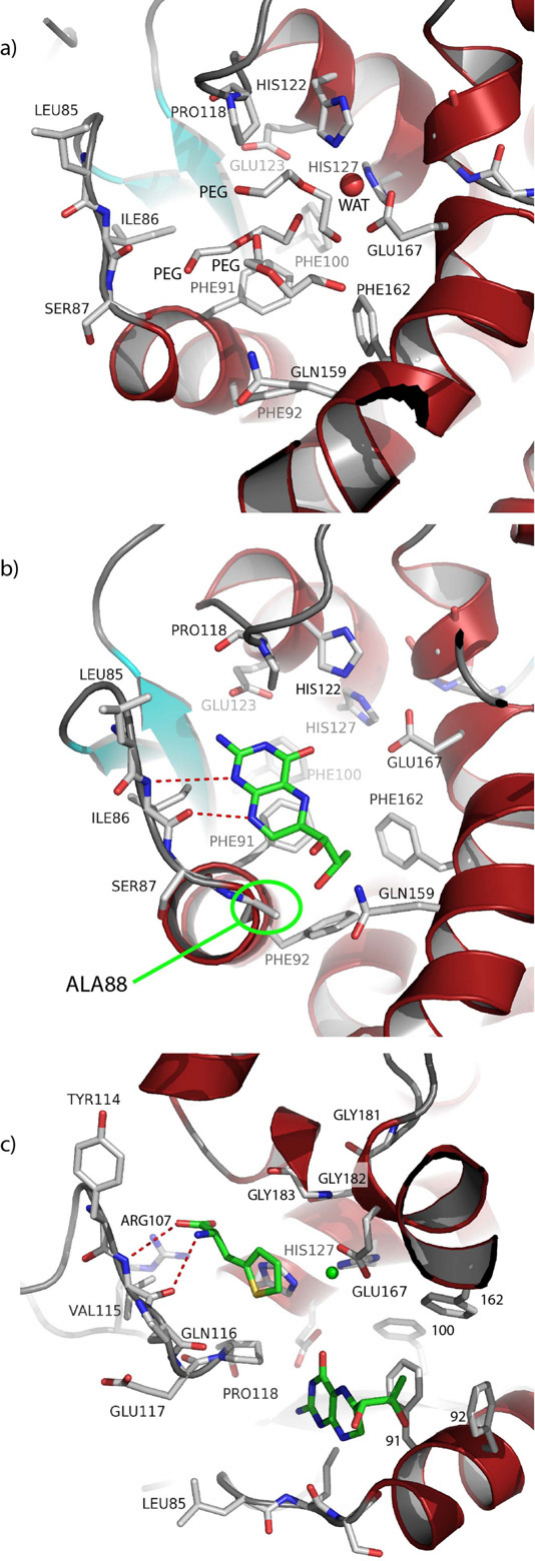
The lpPAH active site. (a) The active site of apo-lpPAH with three polyethylene glycol (PEG) molecules and one water molecule (WAT, in red). (b) lpPAH with modeled BH_2_ (green), and (c) with substrate analogue THA, BH_2_ and Fe^2+^ (green) modeled from hPAH-BH_4_-THA (PDB 1MMK). (For interpretation of the references to colour in this figure legend, the reader is referred to the web version of this article.)

The final lpPAH model refined to an *R*-factor of 27.6% and an *R*-free value of 30.2% (Table 1), which are slightly high but can be explained by the disorder found for chains C and D. The final lpPAH model is most complete for chains A and B, including residues Val8-Asp259 (totally 252 residues for both chains), with lowest mean *B*-factors, whereas chains C (232 residues) and D (237 residues) have higher mean *B*-values and many disordered residues left out of the final model (Table 1, Fig. 1a). The observed differences between the chains can be explained from the symmetry contacts that chains A and B make with one another, whereas chains C and D are facing water channels with less symmetry-related protein–protein interactions. In the active site of lpPAH extra difference electron density was observed and interpreted as three polyethylene glycol (PEG; C_4_H_10_O_3_) molecules (chains A and B) (Fig. 2a).

The catalytic domain of other non-heme iron- and BH_4_-dependent aromatic amino acid hydroxylases (AAAHs) has a mixed α/β fold, also found for the reported lpPAH structure. The root mean square deviation (RMSD; www.ebi.ac.uk/msd-srv/ssm/) of lpPAH compared to other structures are: 1.2 Å to cpPAH (both PDBs 2v27, 2v28 for 252 CA-atoms), 1.3 Å to cvPAH (both PDB 1LTZ/1LTU for 233/232 CA-atoms) and 1.5 Å to hPAH-BH4 (PDB 1J8U for 193 CA-atoms).

### 2.2 Dimerization and intersubunit interactions

In agreement with the dimeric nature of lpPAH in solution [8] there are two dimers in the asymmetric unit, formed by either chains A and D or chains B and C (Fig. 1a). Both dimeric interfaces have similar size and the same residues are involved, thus only the AD interface will be described. Here there are strong ionic interactions (<4 Å) involving Asp198A-Arg247D, Asp228A-Lys172D, Arg247A-Asp198D and Lys172A-Asp228D. There are two additional hydrogen bonds from A to D and D to A involving Arg202 O to Leu204 N and one longer (3.6 Å) polar interaction from Ile199 O to Arg212 NH1 (Fig. 1b). Totally there are 8/7 hydrogen bonds, 4/3 ion pairs <4 Å and additional 3/3 ion pairs 4–6 Å for the AD/BC dimers (Table 2).

Upon dimer formation 21,100 Å^2^, i.e. about 30% of the accessible surface area (ASA) for each monomer, is buried (Table 2). Compared to other functional dimers, this buried ASA is e.g. similar to that in isocitrate dehydrogenase from the psychrophilic bacterium *Desulfotalea psychrophila* [19] and larger than in alkaline phosphatase from the antarctic bacterium TAB5 and in three other alkaline phosphatases [20]. Significantly, the lpPAH dimer interface is very different from that of dimeric truncated hPAH (residues 103–428) [21], since in the latter the C-terminal residues (411–424 in hPAH) form two β-strands involved in the dimerization (Fig. 1c). Also, while in hPAH (residues 103–428) the dimer interface is at the (left) side of the catalytic domain (Fig. 1c), the lpPAH dimer is formed at the top of chain A, with different residues involved in dimerization. In lpPAH, hydrogen bonds (Arg202 O to Leu204 N) bind one β-strand (residues 201–204) from chain A to the same strand from chain D, and these residues, together with ion-pair forming residues 172, 198, 247 and 228, are located towards the middle of the protein chain. This differs from the truncated hPAH dimer where the interactions only involve C-terminal residues (Fig. 1c) [21]. Furthermore, the buried ASA of 519 Å^2^ (3.7% of ASA per monomer) measured in the hPAH dimer interface, is much smaller than in the lpPAH dimer.

In addition to the ionic interactions, which appear to seal the lpPAH dimer together, the interacting interface is composed of the β-strands Val201-Arg202-Ile203-Leu204-Phe205 and Val223-Tyr224-Phe225-Val226-Ile227 from each subunit, and the adjacent hydrophobic C-terminal coils (Glu251-Asp259) where the two Tyr257 residues establish an important stacking interaction (Fig. 1d). The unique dimeric arrangement in lpPAH, and notably the presence of these hydrophobic/aromatic interactions, seems to be an important determinant of the high thermal stability of the enzyme [8] (see below).

### 2.3 Iron dependence

Even when excess iron is added during protein expression, recombinant lpPAH is purified from *Escherichia coli* as an apo (metal-free) enzyme (0.07 ± 0.03 mol Fe(II)/mol subunit) [8]. Also, when activity of the native enzyme is measured in lysates of *L. pneumophila*, addition of 100 μM Fe(II) in the assay gives several-fold higher activity [8], indicating that the active site of lpPAH is sub-saturated with catalytic iron intracellularly. A comparison of LpPAH to cpPAH (PDB 2v27), cvPAH (PDB 1LTV) and hPAH-BH_4_ (PDB 1J8U), identifies the iron-binding residues in lpPAH as His122, His127 and Glu167. In lpPAH (chain A) a water molecule is found at the similar position as Fe(II) in other PAHs in the metal-bound holo-form (Fig. 2a). From the observed electron density it is clearly a solvent molecule and not a metal ion, in agreement with lpPAH being isolated as an apoenzyme [8].

### 2.4 The BH_4_ cofactor and substrate binding sites

Available structures of hPAH complexed with BH_4_ (or BH_2_) either alone or together with the THA substrate analogue ((hPAH-BH_4_ (PDB 1J8U); hPAH-BH_4_-THA (PDB 1MMK) and cvPAH-BH_2_ (PDB 1LTZ)) provide a frame for comparative functional analyses. lpPAH shows a *K*_m_(BH_4_) of 125 ± 25 μM, which indicates lower affinity for the cofactor compared to hPAH (*K*_m_(BH_4_) = 15–29 μM) [22] and cvPAH (*K*_m_(BH_4_) = 15–21 μM) [16,23]. One structural explanation for the lower affinity is that lpPAH cannot provide the same number of hydrogen bonds upon complex formation with BH_4_ as cvPAH (from Asp104) and hPAH (from Ser251) due to Ala88 (Fig. 2b). The side chain of Ala88 in lpPAH is hydrophobic and cannot make a hydrogen bond to O-1’ of BH_2_/BH_4_. Still, lpPAH should be able to make two hydrogen bonds from both Ile86 O and N as observed in hPAH-BH_4_ and cvPAH-BH_2_, as shown in Fig. 2b where BH_2_ is modeled. lpPAH has nevertheless higher affinity for BH_4_ than cpPAH (*K*_m_(BH_4_) = 300 μM) [15] where Phe88 (Ala88 in lpPAH) might sterically hamper the cofactor binding, thus explaining the poor binding of BH_4_ to the cold-active cpPAH.

The enzyme kinetics of lpPAH also indicated a low affinity for its substrate, with a *K*_m_(l-Phe) = 735 ± 50 μM [8] compared with a *K*_m_(l-Phe) = 59 ± 10 μM for cvPAH [16] and *K*_m_(l-Phe) = 60 μM for truncated hPAH (residues 103–428) [24]. We studied the structure for possible residue determinants of the low affinity for l-Phe in lpPAH. The substrate binding-site is best characterized in the crystal structure of truncated dimeric hPAH bound to two substrate analogs l-norleucine (PDB 1MMT) and 3-(2-thienyl)-l-alanine (THA; PDB 1MMK) [25]. When modeling THA into lpPAH the conserved Arg107 interacts with the carboxyl group of l-Phe (Fig. 2c). On the other hand, Tyr114 in lpPAH, which is conserved in all PAHs, is not in the right orientation to contribute in a similar way as in hPAH to the hydrophobic binding site [25], although rotation into another side-chain rotamer might improve the l-Phe binding properties (Fig. 2c). Other adjacent conserved residues that promote l-Phe binding are Pro118, His122, Trp163 and Phe168 where the two latter are found opposite to Pro118. hPAH has a proline residue where lpPAH has Gln116. There are three consecutive glycines (Gly181, Gly182, Gly183) in lpPAH corresponding to Gly, Ala and Gly in hPAH and in cvPAH. These glycines are also found in cold-active cpPAH which has low substrate affinity ([S0.5](l-Phe) = 1.1 ± 0.1 mM) [15]. Assuming that the current orientation is maintained in l-Phe-bound lpPAH, the three consecutive glycines (Gly181, Gly182, Gly183) and Tyr114, might explain the observed relatively low affinity of this enzyme for l-Phe binding.

### 2.5 Thermal stability of lpPAH

Characterization of the thermal stability of iron-free apo-lpPAH both by circular dichroism and differential scanning calorimetry (DSC) has revealed a thermostable enzyme with *T*_m_ = 79 ± 0.5 °C [8], which is much higher than the *T*_m_-values measured for PAH from eukaryotes or other mesophilic bacteria studied so far, clustering around 55 °C [9,26,27]. In fact, lpPAH shows a higher *T*_m_-value than the apo form of PAH from the thermophile *Chloroflexus aurantiacus* (caPAH; *T*_m_ ∼ 64 °C) [12]. Moreover, similar *T*_m_-values are measured for the Fe(II)-bound holoenzymes of both lpPAH (*T_m_* ≃    80 °C) and thermophilic caPAH since the binding of Fe(II) seems to be a large stabilization determinant for caPAH [12] whereas it has almost no effect on the stability of lpPAH [8]. Thus, it seems that specific structural determinants are related to the intrinsic stability of apo-lpPAH, and we notably focused on specific clusters of ionic residues, hydrophobic residues including aromatic clusters, and dimerization.

### 2.6 Polar interactions and amino acid sequence content

As hydrogen bonds and ion-pair content appear to be related to protein thermostability and thermal adaptation, see e.g. [28,29], we analyzed these interactions in lpPAH (Table 2) and compared them with those in cpPAH, cvPAH and hPAH [15]. The lpPAH amino acid sequence has 45%, 38% and 19% sequence identity to cpPAH (*T*_m_ = 52 °C) [15], cvPAH (*T*_m_ = 64 °C) [27] and hPAH (*T*_m_ = 53 °C) [15], respectively (Fig. S1; Supplemental information). lpPAH has 0.84–0.88 hydrogen bonds per residue (Table 2). Comparatively, cpPAH was found to have a higher number of hydrogen bonds per residue (0.981 for holo-cpPAH, PDB 2V27, and 0.951 for apo-cpPAH, PDB 2V28) [15]. It is however important to keep in mind that these numbers are dependent on resolution of the X-ray structure and refinement restrains. Furthermore, for many thermophilic enzymes, extended ion-pair clusters are shown to explain their high thermostability (see e.g. [28,29]). However, for lpPAH both the total number and the number of ion-pairs per defined residue are low (0.017–0.052; Table 2) and the largest ion-pair cluster (<4 Å) comprises only three residues. Thus, from the current lpPAH structure the hydrogen bonds and salt-pairs do not seem to explain the high thermotolerance of this enzyme.

But when analyzing the amino acid sequence, the high Arg/(Arg + Lys) ratio (0.67/0.39/0.61/0.44 in lp/cp/cv/hPAH), the low number of Gly residues (10/13/14/15 in lp/cp/cv/hPAH) and the Pro content (14/12/14/12 in lp/cp/cv/hPAH), might all contribute to the thermostability in lpPAH compared to other PAHs, as inferred from comparative analyses of thermophilic proteins versus mesophilic and psychrophilic orthologs aiming to reveal mechanisms of temperature adaptation [15,30]. While arginine residues appear to be superior to lysines to withstand high temperatures, fewer glycines and additional prolines are associated to a reduction in conformational flexibility [30–33].

### 2.7 Aromatic interactions and the unique dimerization interface

One striking feature in lpPAH is an extended aromatic cluster protruding from the conserved active site residues Phe91, Phe92 (Leu in hPAH), Phe100, Phe139 (Tyr in cp, cv, hPAH) and Phe162. In lpPAH the cluster is extended by residues Tyr143 (Tyr in cp, cvPAH), Phe205 (Phe in cp, cvPAH), Phe211, Phe236 and Phe244, and enclosed by Pro207 (Fig. 1e). In total there are 10/7/7/4 aromatic residues in lp/cp/cv/hPAH in this aromatic cluster found in one single lpPAH chain (Table 3).

There is another aromatic cluster in lpPAH (Trp28, Phe32, Phe47, Phe121, Phe125) lined by Pro62 and Pro129 which is similar in the compared PAHs except hPAH which has one Cys (Phe123 in lpPAH). Structures of the other aromatic amino acid hydroxylase family members tryptophan hydroxylase (PDB 1MLW, 3E2T, 3HF6) and tyrosine hydroxylase (PDB 1TOH, 2TOH, 2XSN) also contain a high number of aromatic residues (Phe, Tyr, Trp, His; data not shown). For the compared PAH structures the number of phenylalanine residues is relatively high in lpPAH (20/16/17/22 in lp/cp/cv/hPAH), and many of the additional aromatic residues are in the described clusters. For hPAH there are 326 residues in the characterized dimeric construct (Gly103-Gln428) and the Phe content is lower (6.7%) than in lpPAH (7.4%) (Table 3). In general, aromatic residues can form stacking pi–pi interactions both in parallel and perpendicular fashion when their ring centers are closer than 7 Å [34,35]. Mutation of one central aromatic residue in isocitrate dehydrogenase from the thermostable *Thermotoga Maritima* decreased the melting temperature by 3.5 °C [28]. Thus for lpPAH these two clusters probably contribute to its high thermal stability.

Furthermore, a third relevant aromatic interaction is formed by Tyr257 in one subunit stacking with the same residue in the adjacent subunit in the dimer. The two Tyr257 residues are held in correct orientation by Pro255 and Pro256 (unique to lpPAH; Fig. S1) and face the corresponding residues in the other chain (Fig. 1d). Phe258, which follows Pro255, Pro256 and Tyr257, is also specific to lpPAH and contributes to the aromatic/hydrophobic character of the most C-terminal part of the dimerization area. This aromatic cluster is thus essential to the formation of the dimerization interface in the enzyme from *L. pneumophila*. This interface is unique among the aromatic amino acid hydroxylases reported to date, and covers a larger area and implies a much larger number of intersubunit interactions than the dimerization motif in the mammalian enzymes (Fig. 1c and see above). Furthermore, lpPAH is the only reported dimeric bacterial PAH, which strongly points to oligomerization as a mechanism for increasing the thermal stability in the bacterial scaffold, as suggested for other proteins (for a review see [31]).

### 2.8 Effect of salt on the thermal denaturation

**Fig. 3:**
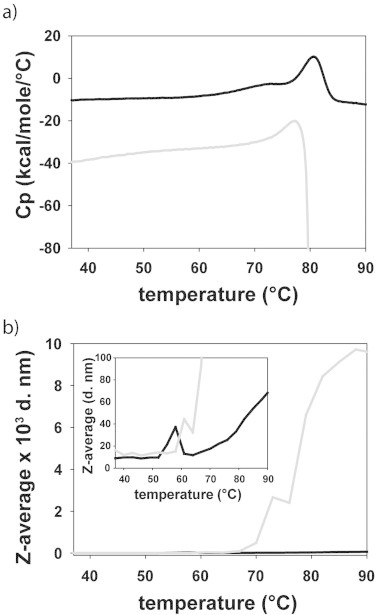
Effect of salt on the thermal denaturation and aggregation of lpPAH. Thermal denaturation was monitored by (a) DSC and (b) DLS. lpPAH (30 μM subunit) was heated in 20 mM Na-Hepes, pH 7.0 (black lines) and in the same buffer with 200 mM NaCl (grey lines). The scan rate was 1 K/min in DSC measurements (a) and the average size of the particles (*Z*-average) was estimated by scattering intensity measurements monitored every 3 °C after an equilibration time of 1 min (b).

In order to obtain thermodynamic insights on PAH from *L. pneumophila*, and on the structure-energetics correlations, we investigated the thermal denaturation of the enzyme both in the absence and the presence of salt. As we have previously shown by differential scanning calorimetry (DSC), the unfolding of lpPAH at pH 7.0 in the absence of NaCl provides an endothermic transition with *T*_m_ = 79 ± 0.5 °C with a calorimetric enthalpy change (Δ*H*) = 169.9 ± 0.2 kcal/mol ([8] and Fig. 3a). The theoretical prediction of Δ*H* at the *T*_m_ (Δ*H*_79_) obtained by energy calculations using the crystallographic structure provides a higher enthalpy change (Δ*H_79_* = 216/218 kcal/mol for chains AD/BC; Table 4), in agreement with the irreversible thermal denaturation of lpPAH and a partially structured denatured state. Actually, CD analysis at 100 °C have shown an increased negative ellipticity at 216 nm [8], indicating that the remaining structure in the denatured state includes β-structure, which might result from intersubunit formation of cross-β interactions [36].

When the thermal denaturation takes place in the presence of 200 mM NaCl, macroscopic aggregation is clearly observed at the end of the thermal transition, which is in addition less endothermic and very distorted by the aggregation associated exothermic process, hindering the determination of the *T*_m_ and Δ*H* at those conditions (Fig. 3a). Still, the apparent *T*_m_ with salt is lower than without (Fig. 3a). This stimulating effect of salt on aggregation might be associated to the special dimerization area, including high content of β-structure and intersubunit hydrophobic interactions surrounded by several ionic pairs (Fig. 1f). Hence, the presence of salt could initially favor the separation of the intersubunit ionic bonds and then reinforce the attractive hydrophobic interactions and enhance aggregation, as also inferred in studies of aggregation-prone proteins in different solvents including salt-free water (see e.g. [37]).

Further insights on the salt effect on the aggregation propensity and type of thermal-induced aggregation of lpPAH were obtained by thermal dependent dynamic light scattering (DLS). As shown in Fig. 3b, the onset of aggregation is retarded when the enzyme is heated in buffer without salt, and the size of the aggregates are >100-fold larger, consistent with the aggregates in salt being visible to the naked eye.

The special architecture of lpPAH at the intersubunit region, with a central core of β-strands establishing intermolecular hydrophobic interactions and surrounded by ionic pairs, indicates that the central area might be most prone to aggregate in a β-type of interaction. We analyzed the aggregation propensity of the protein using the TANGO algorithm [38], which identified residues 163–168 and 225–229 as those having the highest propensity to denaturation. While residues 225–229 indeed comprise the β-strand at the intersubunit interface in the dimer (Fig. 1f), residues 163–168 are located on the last turn of an α-helix consecutive to the former strand, and well oriented to form a cross-β-aggregation upon a local conformational change in the helix. Serpins and prion proteins are prototype of this kind of aggregation [39], but it has also been found in other proteins that do not form amyloid fibers upon aggregation [36].

### 2.9 Physiological relevance of high thermal stability

It is not known whether thermal stability extends to other proteins from *L. pnemophila*. To our knowledge, lpPAH is the only *L. pneumophila* protein for which the denaturation temperature has been determined. Although it is not obvious why an enzyme from *L. pneumophila* would withstand temperatures well above normal growth-conditions, it is interesting that *L. pneumophila* is able to enter a dormant, viable but non-culturable (VBNC) state in response to low nutrient-availability and other environmental stress conditions [18]. These bacteria can be resuscitated upon entering a permissive amoebal host and continue both growth and pathogenicity [40,41]. It will be interesting to investigate if high thermostability is a general trait of *L. pneumophila* proteins or if it is a special property of some few, including PAH. In this later case it is tempting to speculate if lpPAH could be preserved during VBNC due to a beneficial role upon resuscitation.

### 2.10 Conclusion

The presented crystal structure of thermostable phenylalanine hydroxylase from *Legionella pneumophila* (lpPAH) showed a dimeric structure with the unique Tyr257 interacting with the same residue in the adjacent subunit, stabilized by the adjacent Pro255, Pro256 and Phe258 residues. The unique dimeric interface include ion pairs and aromatic interactions, and two additional aromatic clusters per monomer are the plausible structural determinants of the thermal stability of lpPAH. In the presence of salt (200 mM NaCl), macroscopic protein aggregation was observed, compatible with an effect of salt both destabilizing the ion-pairs at the dimer interface and reinforcing the attractive hydrophobic interactions.

## 3 Material and methods

### 3.1 Overexpression and purification of lpPAH

Recombinant wild-type (wt) lpPAH was overexpressed in *E. coli* strain BL21-Codon Plus(DE3)RIL as fusion protein (His)_6_-ZZ-lpPAH with a Tobacco Etch Virus (TEV)-cutting site between the His-tag and ZZ carrier as described [8]. The (His)_6_-ZZ-lpPAH fusion protein was cut with (His)_6_-tagged TEV protease overnight at 4 °C and the isolated lpPAH was obtained by collecting the flow-through from a TALON column, followed by buffer exchange in a desalting (PD-10) column into 20 mM Hepes, pH 7.0 and 200 mM NaCl.

The protein concentration of lpPAH was measured spectrophotometrically using the extinction coefficient *ɛ*_280_ = 1.20 (mg/mL)^−1^ cm^−1^. The purified protein was stored in liquid nitrogen.

### 3.2 Crystallization, structure solution and refinement

Crystallization experiments on lpPAH were performed with a Phoenix crystallization robot (Art Robbins Instruments), at room temperature in 96-well format with MRC plates. Volumes used were 60 μl reservoir solution per well, and drops from 0.5 μl well solution plus 0.5 μl protein solution with 15–21 mg/ml protein in sitting drop experiments. Initially 480 different in-house made conditions were screened followed by optimization around several successful conditions. Still, addition of glycerol, sugars, low molecular weight polyethylene glycol (PEG) and change in pH and precipitant concentration, only improved the crystal quality slightly. The largest crystals grown had a protein concentration of 15–17 mg/ml and with reservoir solutions containing 1.6 M NaK_2_PO_4_ and 4% (v/v) PEG 400. The crystals were cryo protected in 1.6 M NaK_2_PO_4_ and 27% etylene glycol, and then flash frozen in liquid nitrogen.

X-ray data for lpPAH was collected at Bessy, BL 14.1 at 100 K with a wavelength of 0.91841 Å, 6 s exposure, 0.25° oscillation per frame, and in total 103° of data was used in the final data set. The data was integrated and scaled with the program XDS [42] and structure factors were obtained using TRUNCATE [43]. The lpPAH structure was solved by molecular replacement (MR), with a homology model made from *Colwellia psychrerythraea* 34H PAH (cpPAH; PDB 2V27; 45% sequence identity) [15] as search model with the program PHASER [44]. Three molecules were first identified from the MR solution and inspection of the electron density identified the fourth molecule that was further down in the MR solution list.

The structure was refined with REFMAC5 [45] and manually rebuilt in WinCoot [46]. Translation Libration Screw-motion (TLS) refinement was utilized due to disorder in some of the chains. Phenix was also tried as refinement program but it did not improve the statistics nor the electron density maps.

In this paper we have compared our lpPAH structure to cpPAH (PDB 2V27) [15], cvPAH-BH_2_ (PDB 1LTZ) [47], hPAH-Fe(II)-BH_4_ (PDB 1J8U) [25], hPAH-BH_4_-thienylalanine (THA) (PDB 1MMK) [48] and hPAH (PDB 1PAH) [21]. hPAH in these structures is a dimeric truncated form including residues 103–428.

### 3.3 Differential scanning calorimetry (DSC)

DSC was performed using a MicroCal VP-DSC microcalorimeter (GE Healthcare). A sample of 30 μM subunit lpPAH in degassed 20 mM Na-Hepes, pH 7.0, with or without 200 mM NaCl, as indicated, was heated from 37 to 90 °C using a scan rate of 1 K/min. A buffer–buffer reference trace was subtracted and the data was normalized with respect to concentration to obtain excess heat capacity (*C*_p_) as a function of temperature using the MicroCal-enabled Origin 7.0. software. These curves were then analyzed to determine the midpoint denaturation temperature (*T*_m_) and the calorimetric enthalpy change (Δ*H*) for the unfolding transitions.

### 3.4 Dynamic light scattering (DLS)

Thermally induced aggregation of lpPAH was measured by DLS using a Nanosizer S (Malvern Instruments, Sweden) equipped with a He-Ne laser (633 nm) and a fixed 173° back scattering angle. 30 μM subunit lpPAH in 20 mM Na-Hepes, pH 7.0, with or without 200 mM NaCl, was heated from 37 to 90 °C. The average size of the particles (*Z*-average) was estimated by scattering intensity measurements and monitored as a function of temperature with measurements every 3 °C after an equilibration time of 60 s.

### 3.5 Structure-based theoretical unfolding enthalpy values and analysis of aggregation propensity

Calculation of the theoretical unfolding heat capacity change (Δ*C*_p_) and enthalpy change at 60 °C (Δ*H*_60_) was performed using the structure–energetics relationships developed by Freire and co-workers [49], as explained [26], based on the apolar and polar accessible surface area (ΔASA_ap_ and ΔASA_p_, respectively). ΔASA_ap_ and ΔASA_p_ were calculated by Getarea [50] using the crystal structure of dimeric lpPAH (either chains A and D or chains B and C which form two unique dimers in the asymmetric unit).

The TANGO algorithm (http://tango.crg.es/) [38] was used to predict regions involved in β-type aggregation in dimeric lpPAH.

## Protein structure Accession numbers

Coordinates and structure factors for the lpPAH structure have been deposited in the PDB with accession code 4BPT.

## Author contributions

Conceived and designed the experiments: H.-K.S.L., M.I.F., A.M. Performed the experiments: H.-K.S.L., M.I.F. Analyzed the data: H.-K.S.L., M.I.F., A.M. Wrote the paper: H.-K.S.L., A.M.

## Figures and Tables

**Table 1:** X-ray data collection and refinement statistics for lpPAH.

X-ray statistics	lpPAH
PDB entry	4BPT
Beamline	Bessy, BL14.1
Space group	*P*2_1_
Unit cell	*a* = 90.32 Å
	*b* = 60.12 Å
	*c* = 124.04 Å
	*β* = 94.07°
Resolution (Å)	25–2.5
(highest bin)	(2.64–2.5)
Wavelength (Å)	0.91841
No. of unique reflections	41 892 (6 267)
Multiplicity	2.3 (2.3)
Completeness (%)	90.9 (94.0)
Mean (<*I*>/<*σ*_I_>)	8.6 (2.2)
*R*-sym (%)^a^	8.4 (39.4)
Wilson *B*-factor (Å^2^)	41.8

*Refinement*	
Resolution (Å)	10–2.5
*R*-factor (all reflections) (%)^a^	27.64
*R*-free (%)^a^	30.20
No. of atoms	8156
No. of water molecules	162
No. of other molecules	6 PEG
No of residues chain A/B/C/D	252/252/232/237
R.m.s.d. bond lengths (Å)	0.018
R.m.s.d. bond angles (°)	2.08
Average *B*-factor (Å^2^)	
All atoms	47.2
Protein (chain A/B/C/D)	29.8/32.2/67.8/63.0
PEG/Water molecules	26.2/47.4
Ramachandran plot:	
Most favored regions (%)	91.1
Additionally allowed regions (%)	6.4
Disallowed regions (%)	2.5

a *R*_sym_ = (∑*_h_*∑*_i_* | *I*_i_ (*h*) − 〈*I*(*h*)〉|)/(∑*_h_*∑*_I_I*(*h*)), where *I*_i_(*h*) is the *i*th measurement of reflection *h* and <*I*(*h*)> is the weighted mean of all measurements of *h*.

**Table 2:** Structure and dimer interaction analysis of lpPAH. The calculations are done for the residues in the crystal structure only, as indicated.

	lpPAH
No. of res. in chain A/B/C/D	252/252/232/237
No. of H-bonds per residue in^a^	
Chain A	0.881
Chain B	0.865
Chain C	0.853
Chain D	0.844
Ion pairs	
No. of <4 Å Chain A/B/C/D	13/10/4/8
No. of 4–6 Å Chain A/B/C/D	10/12/5/6
No. of ion pairs per residue^a^	
<4 Å Chain A/B/C/D	0.052/0.040/0.017/0.034
*Dimeric interface*	
No. of H-bonds^b^	
Chain A to D	8
Chain B to C	7
No. ion pairs <4/6 Å	
Chain A to D	4/2
Chain B to C	3/3
Accessible surface area (ASA) of dimer (Å^2^)	
Chain A and D	21,150
Chain B and C	21,065
Buried intersubunit surface (% of dimer)	
Chain A to D	30.2
Chain B to C	30.4

a In crystal structure.

b Including ionic interactions.

**Table 3:** Residue content analysis. (a) Residues in an aromatic cluster and (b) number of Phe (F), Tyr (Y), Trp (W) and His (H) residues in lpPAH, cpPAH, cvPAH and truncated hPAH.

Residue No.	lpPAH	cpPAH	cvPAH	hPAH
(a)				
91	F	F	F	F
92	F	F	F	L
100	F	F	F	F
162	F	Y	Y	Y
139	F	Y	Y	Y
143	Y	Y	Y	I
205	F	F	F	L
211	F	L	M	A
236	F	I	A	
244	F	L	L	
(207)	(P)	(I)	(L)	(L)
No. of aromatic residues in cluster	10	7	7	4
(b)				
	lpPAH	cpPAH	cvPAH	hPAH (103–428)
In Gene (no. aa)	272	267	297	326
No. of F	20	16	17	22
No. of Y	13	12	12	20
No. of W	4	4	7	3
No. of H	4	5	6	10
Total No. (%) of F, Y, W, H	41 (15.1%)	37 (13.9%)	41 (14.1%)	55 (16.9%)

**Table 4:** Structure–energetics correlations. The theoretical unfolding changes in heat capacity (Δ*C*_p_) and enthalpy at 60 °C (Δ*H*_60_), and at 79 °C (the *T*_m_-value for lpPAH) (Δ*H*_79_), calculated from the changes in apolar and polar accessible surface area (ΔASA_ap_ and ΔASA_p_) based on the crystal structure of dimeric lpPAH for the A and D or B and C chains, respectively, that form two unique dimers.

	ΔASA_ap_ (Å^2^)	ΔASA_p_ (Å^2^)	Δ*C*_p_ (kcal/K/mol)	Δ*H*_60_ (kcal/mol)	Δ*H*_79_^a^ (kcal/mol)
Chains A and D	12967.4	8183.0	4.2	147.5	218^b^
Chains B and C	12939.5	8125.9	3.7	145.9	216^b^

a The Δ*H*_79_, calculated from Δ*H*_60_ and Δ*C*_p_ using the Kirchhoff equation.

b For comparison, the experimental, calorimetric enthalpy change (Δ*H*), calculated from the DSC scan in the absence of salt (Fig. 3a and Flydal et. al. [8], was 169.9 ± 0.2 kcal/mol.

## Appendix Supplementary materials

**Fig. S1** Sequence alignment of lpPAH, cvPAH, hPAH (Gly103-Gln428) and cpPAH with the secondary structure of lpPAH (top) and cpPAH (bottom; PDB 2V27). The figure was generated by ESPript (http://espript.ibcp.fr/ESPript/ESPript/).
