# Supplementary material for: Structural and thermodynamic insight into phenylalanine hydroxylase from the human pathogen Legionella pneumophila
Source: FEBS Open Bio. 2013 Aug 19;3:370–8. doi: 10.1016/j.fob.2013.08.006 (PMC3821034; doi:10.1016/j.fob.2013.08.006)

# Structural and thermodynamic insight into phenylalanine hydroxylase from the human pathogen *Legionella pneumophila*

**By Hanna-Kirsti S. Leiros, Marte Innselset Flydal and Aurora Martinez**

### Figure S1. Sequence alignment of lpPAH, cvPAH, hPAH (Gly103-Gln428) and cpPAH with the secondary structure of lpPAH (top) and cpPAH (bottom; PDB 2V27). The figure was generated by ESPript (http://espript.ibcp.fr/ESPript/ESPript/)


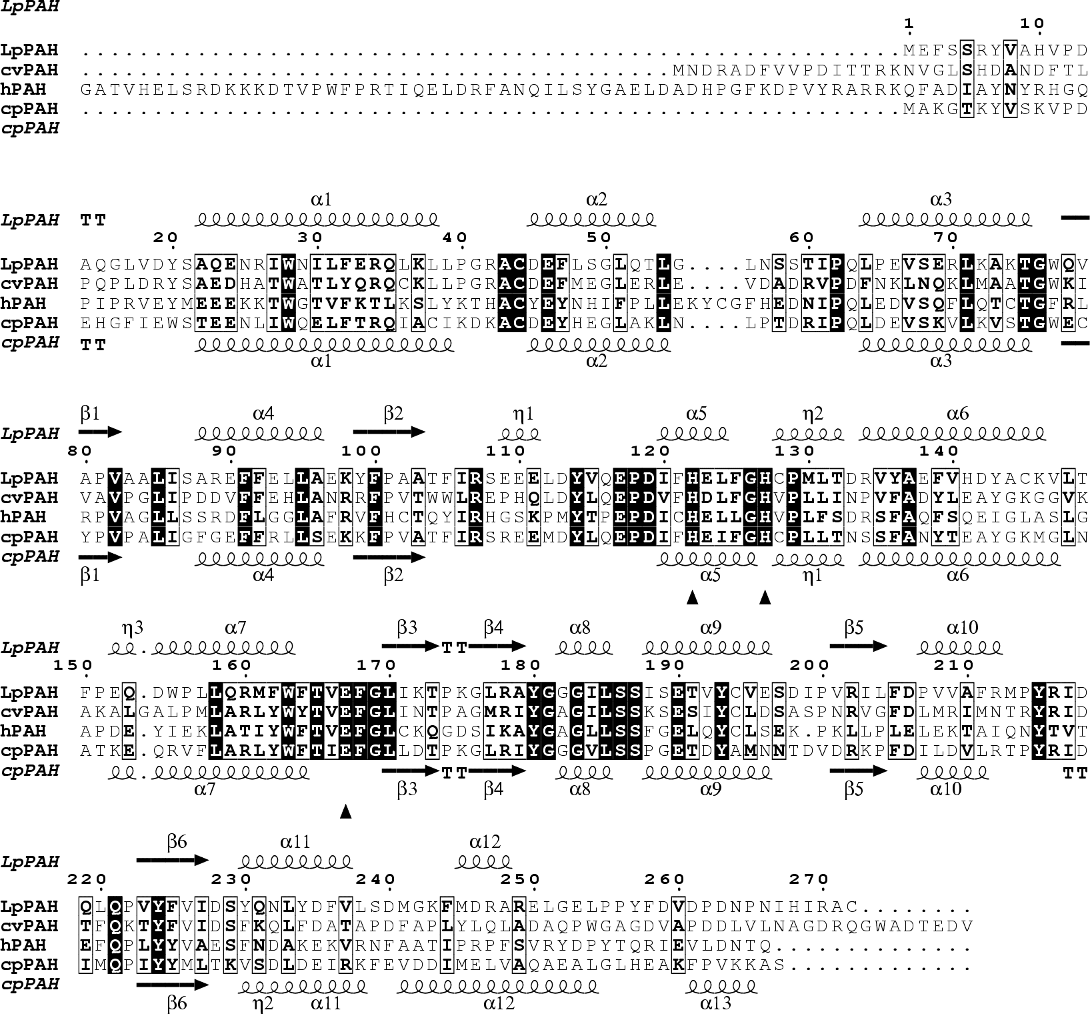

Supplement: Supplementary file 1 — Fig. S1 Sequence alignment of lpPAH, cvPAH, hPAH (Gly103-Gln428) and cpPAH with the secondary structure of lpPAH (top) and cpPAH (bottom; PDB 2V27). The figure was generated by ESPript (http://espript.ibcp.fr/ESPript/ESPript/). [file mmc1.doc]
